# Case Report and Supporting Documentation: Acute Kidney Injury Manifested as Oliguria Is Reduced by Intravenous Magnesium Before Cisplatin

**DOI:** 10.3389/fonc.2021.607574

**Published:** 2021-02-26

**Authors:** Mary Elizabeth Money, Aghiles Hamroun, Yan Shu, Carolyn Matthews, Sara Ahmed Eltayeb, Giuliano Ciarimboli, Christine Noel Metz

**Affiliations:** ^1^Department of Medicine, School of Medicine, University of Maryland, Baltimore, MD, United States; ^2^Department of Medicine, Meritus Medical Center, Hagerstown, MD, United States; ^3^Lille University, Lille University Hospital Center, Nephrology Department, Lille, France; ^4^Institut National de la Santé et de la Recherche Médicale (INSERM), Clinical Epidemiology Team, CESP, Villejuif, France; ^5^Department of Pharmaceutical Sciences, School of Pharmacy, University of Maryland, Baltimore, MD, United States; ^6^Texas Oncology, P.A., Dallas, TX, United States; ^7^Medicine Clinic D, University Hospital Münster, Münster, Germany; ^8^Institute of Molecular Medicine, Feinstein Institutes, Manhasset, NY, United States; ^9^Department of Molecular Medicine, Donald and Barbara Zucker School of Medicine at Hofstra-Northwell, Hempstead, NY, United States

**Keywords:** intravenous magnesium, cisplatin, acute kidney damage, oliguria, hypomagnesemia, nephrotoxicity, ovarian cancer, intra-peritoneal chemotherapy

## Abstract

After more than four decades of post-approval, cisplatin is still an important treatment for numerous cancers. However, acute kidney injury (AKI), defined as significant impairment of renal filtration as discussed below, is the major limiting side effect of cisplatin, occurring in approximately 30% of patients (25–33% after the first course). Cisplatin also damages the kidneys’ ability to reabsorb magnesium in 40–100% of patients, with collateral health risks due to subsequent hypomagnesemia. Multiple methods and drugs have been proposed for preventing cisplatin-induced AKI, including saline infusion with or without mannitol, which has not always prevented AKI and has been found to activate a cellular stress response in renal tubular cells. While numerous reports and trials, as well as the National Comprehensive Cancer Network (NCCN), support premedication with magnesium and hydration, this practice has not been universally accepted. Many clinics administer intravenous magnesium (IV) only after identification of hypomagnesemia post-cisplatin treatment, thus placing patients at risk for AKI and chronic renal loss of magnesium. We present the following case report and additional supporting evidence identifying the immediate effect of IV magnesium prior to intraperitoneal cisplatin for cycle 4 because of documented hypomagnesemia resulting in normalization of oliguria, which had been experienced for the first three cycles. The patient subsequently requested and received IV magnesium before cisplatin for the next two cycles with continuation of normal urinary output. The effect of pretreatment with IV magnesium on urine output following cisplatin has not been previously reported and further supports pre-cisplatin administration. In addition, two recent meta-analyses of clinical trials and pre-clinical research are reviewed that demonstrate effectiveness of magnesium pretreatment to preventing AKI without reducing its chemotherapeutic efficacy. This case report with additional evidence supports the adoption of administration of 1–3 g IV magnesium before cisplatin as best practice to prevent cisplatin induced AKI and hypomagnesemia regardless of patient baseline serum magnesium levels.

## Introduction

Oncologists now have numerous therapeutic agents for various cancers, but cisplatin, the first platinum compound the FDA approved in 1978, continues to be one of the most effective treatments against numerous cancers (1, 2). Cisplatin is highly effective in damaging cancer cell DNA, but its use is restrained by dose-limiting side effects, including AKI, considered to be the most serious toxicity, occurring in approximately one-third of patients (3). Even a single injection of cisplatin may result in a transient episode of AKI in 20–30% of patients (4, 5), which can be missed when measuring only the serum creatinine and blood urea nitrogen. Multiple reviews have discussed the molecular mechanism of AKI induced by cisplatin, which is beyond the scope of this case report (2, 6). Providers in outpatient clinics may not appreciate oliguria as a sign of AKI as manifested by the case report discussed below. Thus, identifying an agent that will prevent or ameliorate this irreversible side effect has been a priority.

The most recognized and followed recommendation to prevent AKI is fluid administration before and after cisplatin, typically with or without mannitol or furosemide (2, 7, 8). Magnesium administered concomitantly with cisplatin has been recommended by Vokes (9) since 1990 to prevent secondary hypomagnesemia due to distal tubular damage (10) and by multiple other clinicians to prevent AKI, (3, 11–16) but has not been established as a standard protocol and was not followed initially for this patient. This review will identify the benefits of this therapy for patients receiving cisplatin, regardless of serum magnesium levels, proposing the adoption of magnesium administration before cisplatin as best practice protocol.

## Case Report

The patient is a 71-year-old board-certified internist who received intraperitoneal (IP) cisplatin and paclitaxel for recurrent ovarian cancer in December 2014.

Background: Stage 3C, poorly differentiated serous adenocarcinoma of the fallopian tube was diagnosed by exploratory surgery at Mercy Medical Center, Baltimore, MD by Dr. Neil Rosenshein in September 2012. Debulking was complete except for 1.5 cm tumor implant on the diaphragm. Chemotherapy with paclitaxel and carboplatin resulted in complete remission by 2013. Background medical history for patient includes: family history for breast cancer (mother and maternal grandmother); non-smoker, history of A–V dura fistula treated conservatively, primary hypothyroidism, allergic rhinitis; BP 112/78, pulse 72, BMI 20.9. Patient was entirely asymptomatic but CA 125 had increased from eight to 19 on 10/20/14 and a PET scan showed 1 cm implant on the right kidney on 10/31/14. Rather than accepting standard chemotherapy, the patient underwent exploratory laparotomy by Dr. Robert Edwards at Magee-Women’s hospital, UPMC, Pittsburgh, PA. No tumor implant was identified, but cell washings were atypical and an intraperitoneal port was placed for IP therapy.

Baseline serum lab included: blood urea nitrogen (BUN) 16, creatinine (Cr) 0.7, and magnesium 2.0 mg/dL (normal range: 1.7–2.2) on 12/26/14. Cisplatin and paclitaxel were administered *via* IP infusion every 21 days for six cycles beginning on 12/29/14 (day 1). Paclitaxel 135 mg/m^2^ in 500 ml of normal saline intravenous (IV) over 3 h was administered on day 1. After 1 L of normal saline IV, cisplatin at 75 mg/m^2^ was given in 1 L of normal saline IP followed by a second liter of normal saline IP, if tolerated. Another liter of normal saline IV was subsequently administered, too. Significant oliguria (concentrated, dark urine estimated at <30 cc h) was observed within 3 h after IP cisplatin. This oliguria and abdominal distention with pain continued for the next 36–48 h despite >2,000 cc oral intake of liquids and an additional liter of normal saline IV the following day.

Because the patient (an internist) recognized the disproportional oliguria, the on-call provider was consulted that evening within 6 h of cisplatin administration, but no action was recommended since the patient still had some urine output. AKI, which manifested as oliguria, was not recognized and stat renal function studies were not ordered. Diuresis had occurred by 1/3/15 when the following tests were obtained prior to having urgent surgery for a fractured wrist due to a fall on ice: BUN 20, Cr 0.74, and magnesium 1.3 mg/dL.

Oral magnesium oxide >500 mg was consumed daily. Intravenous magnesium was not administered unless serum magnesium dropped below 1.5 mg/dL, which was only checked immediately before each cycle of chemotherapy and not in between. On 2/9/15, prior to the next cycle, which had been delayed for 3 weeks due to a wrist fracture, BUN was 22, Cr 0.8, and magnesium 1.8.

The patient continued to experience severe oliguria immediately following cisplatin IP administration, lasting for approximately 48–72 h for the next two cycles, but on day 1 of cycle 4 on 3/25/15, 4 g (32.48 mEq) magnesium sulfate was administered IV prior to chemotherapy for a serum magnesium of 1.0 mg/dL. (Serum magnesium had been 1.6 on 3/2/15). The patient immediately observed normal urinary output on day 2 post-cisplatin and thereafter. In addition, abdomen distention and pain were significantly less than during the prior cycles.

Recognizing this response, the physician-patient completed a literature search regarding the effect of magnesium on cisplatin toxicity and consulted with a scientist who had recently published studies in rodents demonstrating the benefits of pretreating with magnesium to protect against cisplatin-induced AKI (17). Therefore, the patient subsequently requested and received 2 g of magnesium sulfate IV pre-cisplatin (on day 2) for cycles 5 and 6 even though serum magnesium was 1.7 mg/dL. To clearly demonstrate the effectiveness, she recorded the fluid intake and output for day 2, cycle 6: 4,800 cc combined IV, IP, and oral route, with a concomitant urine output of 4,950 cc in 24 h. The patient continued to demonstrate improved tolerance to the IP treatment with the administration of IV magnesium preceding cisplatin.

After completion of the six cycles, on 6/29/15, relevant values were: BUN 19, serum Cr 0.9, and magnesium 1.9. Patient at that time was receiving 1–2 g of IV magnesium weekly and has continued to suffer from persistent hypomagnesemia requiring 600 mg of oral magnesium threonate in divided doses daily to prevent tetany. These effects are presumed due to the irreversible AKI from cycles 1–3 prior to pretreatment with magnesium in subsequent cisplatin cycles. Glomerular filtration rate from 2015 until 2020 has been greater than 59 ml/min and current renal function on 8/27/20 was: BUN 23, Cr 1.04. As a physician, the patient strongly supports this case report to prevent AKI, hypermagnesemia, and the discomfort associated with intraperitoneal cisplatin that was diminished with magnesium preceding cisplatin.

## Discussion

### Overview of Acute Kidney Injury by Cisplatin

According to the Kidney Disease Improving Global Outcomes (KDIGO), AKI is defined as ≥0.3 mg/dL increase in serum creatinine or a 0.5 ml/kg/h decrease in urine output within 48 h; whereas Common Toxicity Criteria for Adverse Events Version 4.0 (CTCAE v4.0) agrees on serum creatinine increase ≥0.3 mg/dL but with no time consideration (5).

Using CTCAE v4.0 criteria, 26.5% of patients experienced AKI after the first round of cisplatin-based chemotherapy in one retrospective study for urothelial cancer, resulting in >40% being unable to receive the planned second round, and 50% reduction in 3-year survival (5). Consequently, mortality is increased among patients with AKI, frequently due to inability to continue chemotherapy as scheduled (18, 19).

Risk factors for renal damage include people who are older, female, African American (20), smokers, have hypoalbuminemia, prior kidney damage, hypomagnesemia, dehydration, and/or have concomitant medical conditions such as diabetes, liver disease and use of angiotensin receptor blockers, angiotensin-converting enzyme inhibitors, diuretic therapy, and non-steroidal anti-inflammatory drugs (16–18).

Cisplatin is freely filtered at the glomeruli but is subsequently absorbed by the proximal tubule cell where it becomes a more potent toxin by multiple enzymatic pathways including gamma glutamyl transpeptidase (GGT), which has the highest activity in the kidneys (2, 6, 21).

However, a consistent amount of cisplatin is secreted from the blood into the urine through the cells of proximal tubules (22). Here, cisplatin uptake is accomplished by the human copper transport protein 1 (Ctr1) and the organic cation transporter 2 (OCT2) (23) located on the basolateral side of the proximal (24) and distal (25) renal tubules, as well as by passive diffusion (26). Concentration in the proximal tubule may be five times greater than the blood (27), with intrinsic damage of the proximal and distal tubules resulting in renal tubular cells death, affecting renal tubular blood flow, decreasing glomerular filtration rate, and preventing reabsorption of magnesium and other electrolytes (2). Excretion from the tubular cells is dependent on multidrug extrusion transporters (MATEs) (6, 28). Although renal tubular cells may recover, fibrotic scarring may occur resulting in chronic kidney disease (CKD) (29).

Bunel et al. (29) in a small human study, identified an increase in urinary biomarkers for acute renal damage within 3 h after cisplatin administration, but a diagnostic rise in plasma creatinine in each patient with AKI was delayed until 3–6 days post-administration, by which time urinary biomarkers had normalized.

## Summary of Studies Demonstrating Effectiveness of Magnesium in Humans

In 2019, two systematic reviews and meta-analysis of therapies directed at prevention of cisplatin AKI were published, both suggesting a benefit with pre-administration of IV magnesium (30, 31) (**Table 1**). Casanova reviewed all placebo-controlled trials published up to 2017 (22 met their criteria), and concluded that 1 g (8 mEq) of IV magnesium before cisplatin reduced AKI (30).

**Table 1:** Published results on the association between cisplatin-induced AKI* and magnesium administration.

Study	Intervention and outcome(s)		Results	P-value
**Hamroun et al**. (31) ***Drugs***	Risk of cisplatin-induced AKI*		**Odds Ratio** [95% Confidence Interval]	
	Magnesium dosage	All dosages confounded	0.24 [0.19; 0.32]	<0.001
		8 mEq	0.23 [0.16; 0.34]	<0.001
		20 mEq	0.13 [0.06; 0.29]	<0.001
		25 mEq	0.28 [0.14; 0.54]	<0.001
**Casanova et al**. (30) ***Eur J Clin Pharmacol***	Risk of cisplatin-induced AKI*		**Odds Ratio** *[95% Confidence Interval]*	
	Magnesium dosage	All dosages confounded	0.22 [0.15; 0.33]	<0.001
	**Modification in serum creatinine levels**		**Mean difference of serum creatinine** *(mg/dL)* *[95% Confidence Interval]*	
	Magnesium dosage	All dosages confounded	−0.19 [−0.34; −0.05]	< 0.001

*AKI, acute kidney injury defined by the 2012 KDIGO-AKI classification.

Hamroun et al. (31) searched Pubmed, Embase, and Web of Science from January 1, 1978, to June 1, 2018, assessing cisplatin AKI as defined by the 2012 AKI-KDIGO classification, which identified stage 1 as either serum creatinine 1.5–1.9 times OR ≥0.3 mg/dL (≥26.5 mmol/L) increase above baseline, or urine output decrease to 0.5 ml/kg/h for 6–12 h; stage 2: serum creatinine 2–2.9 × baseline, or decrease in urine output <0.5 ml/kg/h for × 12 h; stage 3: serum creatinine 3 × baseline or urine output <0.3 ml/kg/h (32). Of 4,520 eligible studies reviewed, 51 articles fulfilled the authors’ selection criteria, which included evaluating 21 different prevention methods. A meta-analysis was only performed on those studies that used magnesium at the same time as the first dose of cisplatin (15 observational involving 1,841 patients), and demonstrated a significant AKI protection for all grades of injury (31). Based upon analysis of the data regarding stage 1 AKI, “25 mEq of magnesium was associated with a significant nephron-protective effect (OR 0.20 [0.12–0.31], with a positive trend test (p = 0.002)” (31).

## Magnesium Prevents Renal Toxicity in Animal Studies: Potential Mechanisms

Like humans, laboratory animals exhibit hypomagnesemia and AKI following serial cisplatin doses. Cisplatin specifically targets the proximal tubules, which comprise a significant portion of the kidneys. The proximal tubules contain a high density of epithelial cells with a large number of mitochondria necessary for providing the critical regulatory (pH balance, absorption, and secretion) and endocrine functions of the kidneys. In mice, organic cation transporters 1 and 2 (OCT 1 and OCT2) located on proximal tubule epithelial cells are considered to be the main transporters involved in cisplatin uptake by the renal proximal tubules (33, 34). Thus, this segment of the nephron is most susceptible to cisplatin-induced AKI (35, 36). Once taken up by the renal epithelial cell, cisplatin mediates its acute toxic effects by enhancing inflammation [*via* the Extracellular Signal Regulated Kinase (ERK) and Signal Transducer and Activator of Transcription 3 (STAT3) signaling and subsequent cytokine production], increasing oxidative stress and by inducing cell death (apoptosis, necrosis, and autophagy).

Animal studies also demonstrate that cisplatin treatment lowers serum magnesium levels (37, 38) and that poor magnesium status enhances cisplatin-induced AKI (17, 37, 39, 40). Although the exact mechanism(s) involved are not completely understood, there is some evidence showing that magnesium absorption is impaired by cisplatin-mediated renal damage, suggesting that magnesium deficiency augments cisplatin uptake (probably *via* OCT2 and Ctr1) and reduces elimination (*via* MATE1) by kidney epithelial cells. Most importantly, the nephrotoxicity of cisplatin can be blocked by early and sustained magnesium supplementation in magnesium-deficient animals, preventing irreversible kidney injury (17, 39). Additional data support that the host’s magnesium status regulates multiple pathways associated with cisplatin-induced AKI, including oxidative stress, inflammation and apoptosis, and early magnesium supplementation protects against cisplatin-induced kidney damage through modulating these pathways (17, 40). Finally, while magnesium deficiency was associated with significantly larger tumors in mice and reduced cisplatin-mediated tumor killing *in vivo*, early magnesium supplementation was shown to protect the kidneys against cisplatin-mediated damage without compromising cisplatin anti-tumor efficacy while additionally potentiating the cytotoxic effect (39, 40). Together, these data strongly support that early magnesium supplementation exerts kidney-protective effects and may improve the anti-tumor efficacy of cisplatin.

## Importance of Magnesium Homeostasis and Health Risks Associated with Hypomagnesemia

Over 300 biological enzymes are dependent on magnesium, the second most abundant intracellular cation, and fourth most abundant cation in the body (41). The normal body contains 22–26 g of magnesium; 52.9% in the bone, 27% in the muscle, 19.3 in soft tissue, 0.5% in red blood cells, and 0.3% in the serum (42).

Although magnesium deficiency is almost always asymptomatic (43), it may lead to long-term health problems including but not limited to: affecting cardiac electrical activity, including sudden death; association with insulin resistance, inhibiting acute phase of insulin release in hyperglycemia; contributing to progression of atherosclerosis by affecting lipid concentrations; hypertension; osteoporosis; increase frequency in renal calculi; reactive airway disease; muscle weakness; and multiple non-specific complaints including: fatigue, anorexia, fibromyalgia, tendonopathy, tetany, and mood alterations (41).

Measurement of the serum magnesium level is not an accurate reflection of total body stores. Renal excretion is predominantly responsible for maintaining serum balance with 70–80% of non-protein bound magnesium being filtered at the glomerulus, 95% of the magnesium in plasma is reabsorbed by the kidneys (60% at the ascending loop of Henle and 10% in the distal tubule, resulting in a loss of only 100 mg) (41, 42, 44).

Although not labeled AKI, the most common evidence of early renal damage is hypomagnesemia, first identified in 1979 (45). Hypomagnesemia may enhance the severity of nephrotoxicity (46). In addition, observational studies in humans, similar to those in animals, have demonstrated that premedication with magnesium prior to cisplatin may reduce the nephrotoxicity of magnesium loss. In 1990, Vokes (9) reported a randomized study of 23 patients treated with cisplatin for head and neck cancer using oral magnesium aspartate hydrochloride either by continuous oral magnesium, with dosage being increased or magnesium supplemented intravenously if unable to be tolerated *versus* intermittent administration only if the serum magnesium level dropped to ≤1.4 mg/dL. All patients receiving intermittent magnesium required magnesium at some point in the study, but 80% of patients receiving continuous magnesium never developed hypomagnesemia in a given cycle. Likewise, Martin et al. (47) demonstrated in 1992 that both intravenous (3 g before each cycle) and oral magnesium supplementation (2 g orally every 8 h, days 2–21 of each cycle) appeared effective in prevention of cisplatin-induced hypomagnesemia in the majority of patients with only mild gastrointestinal side effects observed with the oral group. Vokes concluded that “preventive administration of a magnesium supplement can ameliorate, if not completely eradicate, cisplatin-induced hypomagnesaemia.” (9) Hodgkinson, in 2006, also recommended routine supplementation of magnesium with each cycle of cisplatin to prevent cisplatin-induced hypomagnesemia (48). Most recently, a recent review by Duan supports magnesium administration to prevent AKI in elderly patients receiving cisplatin along with short hydration and amifostine (49).

Intravenous magnesium has been shown to be safe (50) and effective in multiple other medical conditions. It has been used to prevent AKI in contrast-induced nephropathy in primary percutaneous coronary intervention (51) and when administered intraoperatively with major laparoscopic abdominal surgery (52). Magnesium sulfate has been demonstrated to be more effective than anticonvulsants in acute eclampsia and reduces the risk of eclampsia by 50% in pre-eclampsia (41, 53). It has been used for status asthmaticus, torsades de pointes, and a higher concentration of magnesium has been correlated with better survival in chronic kidney disease (54). Lactate clearance has been shown to decrease in critically ill patients with severe sepsis with magnesium supplementation achieving a serum magnesium level near the upper limit of normal.

**Figure 1:**
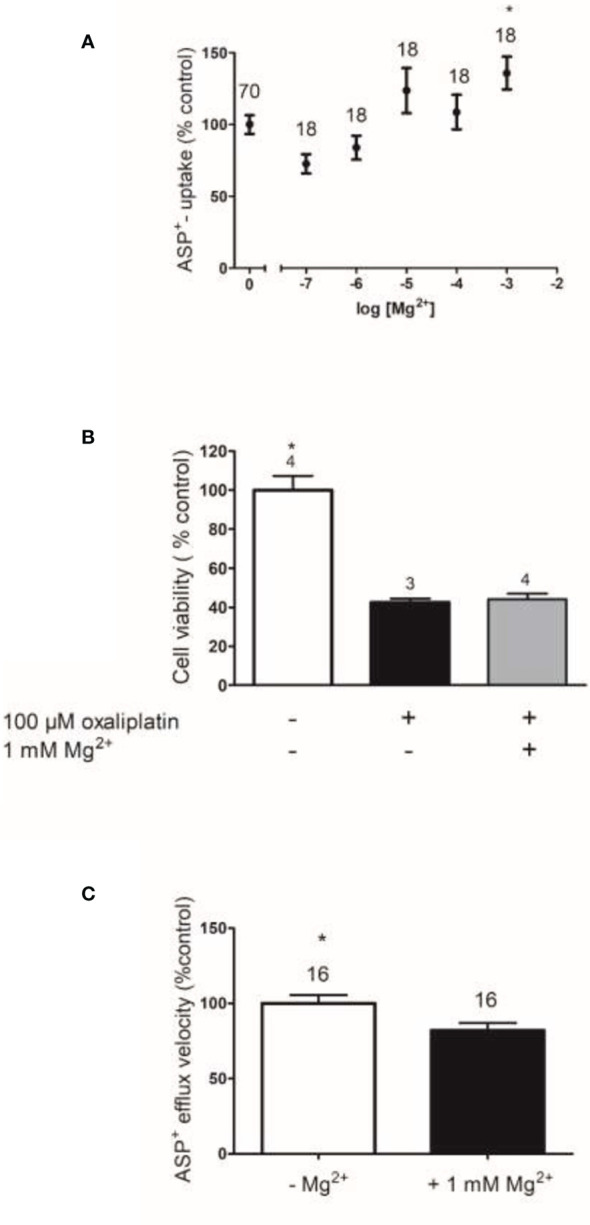
The effects of Mg^2+^ on hOCT2 function **(A)**, on hOCT2-mediated Oxaliplatin toxicity **(B)**, and on hMATE1 function **(C)**. Function of the transporters was measured as uptake (hOCT2) or efflux (hMATE1) of the fluorescent organic cation 4-[4-(dimethylamino)styryl]-N-methylpyridinium (ASP^+^) as described in Wilde et al. (56) and Kantauskaite et al. (57) Panel **(A)** shows the changes of ASP^+^ uptake by hOCT2 stably expressed in Human Embryonic Kidney (HEK) cells in dependence from extracellular Mg^2+^ concentration compared to what was measured in Mg^2+^ absence (mean ± SEM). Only 1 mM Mg^2+^ significantly increased ASP^+^ uptake by hOCT2 (Kruskal–Wallis test with Dunn’s multiple comparison test). The presence of Mg^2+^ stimulates the activity of hOCT2, a transporter mediating nephrotoxicity of platinum derivatives, therefore regulation of hOCT2 by Mg^2+^ cannot explain Mg^2+^ protective effects against cisplatin nephrotoxicity. **(B)** shows the effects of Mg^2+^ supplementation (1 mM) on toxicity of 100 µM Oxaliplatin measured with a 3-(4,5-dimethylthiazol-2-yl)-2,5-diphenyltetrazolium bromide (MTT) assay in hOCT2 overexpressing HEK cells (mean ± SEM). The presence of 1 mM Mg^2+^ does not protect the cells against hOCT2-mediated Oxaliplatin toxicity. Finally, **(C)** shows the changes of ASP^+^ efflux by hMATE1 stably expressed in HEK cells in dependence from extracellular Mg^2+^ concentration compared to what measured in Mg^2+^ absence (mean ± SEM). The extracellular presence of 1 mM Mg^2+^ slightly but significantly decreases the function of hMATE1 (Mann–Whitney-test), which is an efflux transporter for platinum derivatives. Therefore, also a regulation of hMATE1 by Mg^2+^ cannot explain Mg^2+^ protective effects against cisplatin nephrotoxicity. In **(A, C)** the numbers above the columns represent the number of replicates measured in at least three independent experiments. In **(B)** they represent the number of independent experiments.

How exactly IV magnesium prevents AKI when given prior to cisplatin in humans is currently unknown and possibly involves numerous pathways as discussed previously. Although magnesium downregulated the OCT2 transporter and upregulated the MATE transporter, preventing AKI in rats, (55) this was not observed with acute exposure in recent experiments using cells expressing human OCT2 performed by Dr. Ciarimboli (see **Figure 1 A–C**). Therefore, further studies will be necessary to completely understand how magnesium prevents AKI in humans. Other drugs have demonstrated a downregulation of human OCT2 (hOCT2), protecting against AKI, such as carvedilol, (58) while metformin and cimetidine have been competitive substrates for hOCT2. Research has shown the renal protective effects of both of these agents from cisplatin toxicity (59, 60). However, two meta-analyses have both identified that IV magnesium prevents AKI from cisplatin and recommend it over all other agents at this time.

## Conclusion

Currently, there is no dispute regarding the renal toxicity associated with cisplatin. AKI, based on elevation of serum creatinine or decreased urine output, occurs in approximately one-third of all patients receiving cisplatin. Hypomagnesemia occurs in 40–100% of patients following cisplatin and may persist long after chemotherapy completion, reflecting irreversible cisplatin-mediated kidney damage. What has been controversial is the administration of IV magnesium prior to each dose of cisplatin, rather than after inevitable hypomagnesemia is subsequently identified. Compelling recent reviews of human trials and animal studies clearly support the pre-administration of IV magnesium for ameliorating both AKI and hypomagnesemia. Acute cisplatin-induced nephrotoxicity (including AKI and hypomagnesemia) may cause persistent and irreversible kidney impairment, resulting in further health complications.

Hesitancy regarding IV magnesium prior to cisplatin may have originated from the early termination of the Combined Oxaliplatin Neurotoxicity Prevention (CONcePT) in 2007, in which calcium/magnesium was infused before oxaliplatin chemotherapy for colon cancer to prevent neurotoxicity. Although initial data suggested a 52% reduction in oxaliplatin’s killing efficacy following calcium/magnesium administration, (61) this was subsequently reversed in 2008 and Wu, in 2012, published a systematic review concluding that IV calcium/magnesium does not impair oxaliplatin effectiveness (56). In addition, there has been no evidence in any of the trials analyzed that magnesium affected the chemotherapeutic effect of cisplatin, and a higher magnesium level actually potentiated cisplatin chemotherapeutic effect in mice (40). Finally, the OCT2 transporter that is involved in the cisplatin/oxaliplatin uptake and subsequent AKI has not been identified in tumors.

This case report along with supporting documentation provided by both clinical and pre-clinical studies clearly demonstrate the effectiveness of IV magnesium before cisplatin in preventing acute kidney injury manifested by oliguria. We recommend that at least 2 g of magnesium be administered prior to cisplatin since a recent study by Uhm found that 1 g still resulted in 33% of patients developing hypomagnesium (57), while a study by Hase supported the use of 20 mEq/L or approximately 2.5 g. (62)

Therefore, if we are to follow our oath to do no harm, it is imperative that IV magnesium administration with cisplatin become a “best practice” guideline at all oncology centers.

## Data Availability Statement

The original contributions presented in the study are included in the article/supplementary material. Further inquiries can be directed to the corresponding author.

## Ethics Statement

Written informed consent was obtained from the individual(s) for the publication of any potentially identifiable images or data included in this article.

## Author Contributions

MM: concept for paper and case report. AH: review of human studies and comparison of recent meta-analyses, and development of table comparing these. CM: editing and review of paper. YS: review of renal receptors and effect of magnesium on them. CNM: summary of research in animals and editing of manuscript. SE: assistance in experiments using magnesium with human renal transporters GC: development and analysis of experiments using magnesium with human renal transporters. All authors contributed to the article and approved the submitted version.

## Funding

Source for funding: Medical Research Fund Community Foundation of Washington County, Inc 37 S. Potomac Street Hagerstown, MD 21740.

## Conflict of Interest

The authors declare that the research was conducted in the absence of any commercial or financial relationships that could be construed as a potential conflict of interest.
